# Interrogating the Evolutionary Paradox of Schizophrenia: A Novel Framework and Evidence Supporting Recent Negative Selection of Schizophrenia Risk Alleles

**DOI:** 10.3389/fgene.2019.00389

**Published:** 2019-04-30

**Authors:** Chenxing Liu, Ian Everall, Christos Pantelis, Chad Bousman

**Affiliations:** ^1^Department of Psychiatry, Melbourne Neuropsychiatry Centre, University of Melbourne and Melbourne Health, Melbourne, VIC, Australia; ^2^Institute of Psychiatry, Psychology and Neuroscience, King’s College London, London, United Kingdom; ^3^South London and Maudsley NHS Foundation Trust, London, United Kingdom; ^4^Florey Institute of Neuroscience and Mental Health, University of Melbourne, Melbourne, VIC, Australia; ^5^Department of Electrical and Electronic Engineering, Centre for Neural Engineering (CfNE), University of Melbourne, Carlton South, VIC, Australia; ^6^Melbourne Health, NorthWestern Mental Health, Melbourne, VIC, Australia; ^7^Department of Medical Genetics, University of Calgary, Calgary, AB, Canada; ^8^Department of Psychiatry, University of Calgary, Calgary, AB, Canada; ^9^Department of Physiology and Pharmacology, University of Calgary, Calgary, AB, Canada

**Keywords:** schizophrenia, evolution, GWAS, Neanderthal, negative selection

## Abstract

Schizophrenia is a psychiatric disorder with a worldwide prevalence of ∼1%. The high heritability and reduced fertility among schizophrenia patients have raised an evolutionary paradox: why has negative selection not eliminated schizophrenia associated alleles during evolution? To address this question, we examined evolutionary markers, known as modern-human-specific (MD) sites and archaic-human-specific sites, using existing genome-wide association study (GWAS) data from 34,241 individuals with schizophrenia and 45,604 healthy controls included in the Psychiatric Genomics Consortium (PGC). By testing the distribution of schizophrenia single nucleotide polymorphisms (SNPs) with risk and protective effects in the human-specific sites, we observed a negative selection of risk alleles for schizophrenia in modern humans relative to archaic humans (e.g., Neanderthal and Denisovans). Such findings indicate that risk alleles of schizophrenia have been gradually removed from the modern human genome due to negative selection pressure. This novel evidence contributes to our understanding of the genetic origins of schizophrenia.

## Introduction

Schizophrenia is a severe, highly heritable (*h*^2^ = 0.64–0.80) psychiatric disorder that typically emerges in late adolescence or early adulthood (Thaker and Carpenter, 2001; Lichtenstein et al., 2009; van Os and Kapur, 2009). The peak of illness onset differs by sex regardless of culture, definition of onset, and definition of illness, with onset peaking at 15–25 years of age in men and 20–35 years of age in women (Mendrek and Mancini-Marïe, 2016). Aligned with these onset peaks, evidence indicates that schizophrenia patients, particularly males, have reduced rate of reproduction (fitness) compared with non-affected populations (Bassett et al., 1996; Avila et al., 2001). Although it has been reported that fertility among relatives of patients with schizophrenia is increased, a large cohort study and meta-analysis identified that this increase was too small to counterbalance the reduced fitness of affected patients (Bundy et al., 2011; Power et al., 2013). In fact, MacCabe et al. (2009) showed that patients with schizophrenia had fewer grandchildren than in the general population, demonstrating that the reduced reproductivity persists into subsequent generations. This reduction in overall reproduction among those with schizophrenia and their progeny, coupled with high heritability should result in a decrease in schizophrenia according to the evolutionary concept of negative selection. Negative selection results in the purging of deleterious alleles that contribute to traits that reduce fertility. However, the principle of negative selection seems inconsistent with schizophrenia, which is characterized by both high heritability and reduced fertility (Avila et al., 2001) but relatively stable prevalence in the population, suggesting an evolutionary paradox.

Some have attempted to explain this paradox by proposing that risk alleles for schizophrenia at some time in human history conferred evolutionary advantages (i.e., mating success or reproductivity) (Karksson, 1970; Waddell, 1998; Turelli and Barton, 2004; Nettle and Clegg, 2006), while others have attributed the existence of these risk alleles as a price paid for language and development of the social brain (Crow, 1997, 2000). The former evolutionary perspective in schizophrenia has been explained by Nettle (2001), Nettle and Clegg (2006), who suggested that schizotypy characteristics could be linked to intelligence, artistic creativity and thus may positively correlate with mating success. A recent cross-trait analysis of genome-wide association study (GWAS) data supports this notion in that higher polygenic risk scores for schizophrenia predicted creativity (Power et al., 2015). The latter explanation by Crow proposed schizophrenia as a price the modern human paid for achievement of language (Crow, 1997). This idea was subsequently incorporated in the so-called “by product” hypothesis of schizophrenia by Burns (2004, 2006). The by product hypothesis relies on the argument that schizophrenia shares a common genetic basis with the evolution of the social brain, representing the abnormal cortical connectivity that occurred approximately 1 to 1.5 million years ago in our ancestors, archaic humans (e.g., Neanderthals, Denisovans). Other evolutionary theories, such as ancestral neutrality and polygenic mutation-selection balance, have been proposed to explain the evolutionary paradox (Keller and Miller, 2006). However, a consensus has not been reached by evolutionary scientists.

The development of evolutionary genomic tools and the emergence of a critical mass of GWAS data have provided the opportunity to empirically examine the “schizophrenia paradox” and uncover evolutionary mechanisms underpinning the pathogenesis of schizophrenia. Xu et al. (2015) identified the enrichment of schizophrenia SNPs near human accelerated regions (HARs) in the genome that are conserved in primates but have undergone accelerated evolution in humans (pHAR, a type of HARs based on conservation of non-human primates). More recently, Srinivasan et al. (2016) applied a novel evolutionary statistic, the Neanderthal selective sweep (NSS) score, to the largest schizophrenia GWAS dataset (Schizophrenia Working Group of the Psychiatric Genomics Consortium, 2014) and found SNPs associated with schizophrenia were significantly (*p* = 7.30 × 10^−9^) enriched in genome regions that were under recent positive selection. However, recent GWAS findings by Pardiñas et al. (2018) have challenged the notion of selective advantage of schizophrenia risk alleles by demonstrating that these risk alleles have undergone strong background (negative) selection.

**FIGURE 1:**
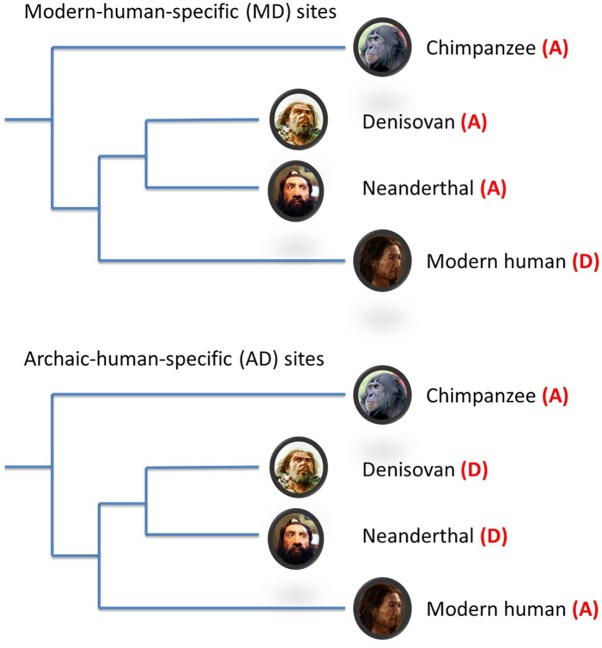
Schematic illustration of modern-/archaic-human-specific site. In the figure, (A) indicates ancestral allele and (D) indicates non-ancestral (derived) alleles. Modern-human-specific (MD) sites are those sites where Denisovans or Altai Neanderthals have the derived allele and the ancestral allele is fixed or appears at a high frequency (>90%) in modern humans. Archaic-human-specific sites are those sites. For each site, the ancestral/non-ancestral state (allele) was determined via a comparison with the chimpanzee genome.

To assist in reconciling the current evidence to date, additional evolutionary genomic markers i.e., modern-human-specific (MD) sites and archaic-human-specific (AD) sites have recently become available (Prüfer et al., 2014; Figure 1). These genomic sites provide an opportunity to further interrogate the schizophrenia paradox and examine in more detail the direction of evolutionary mechanisms on SNPs/alleles associated with schizophrenia after modern humans split from archaic humans. As such, we analyzed the Psychiatric Genomics Consortium (PGC) schizophrenia GWAS data (Schizophrenia Working Group of the Psychiatric Genomics Consortium, 2014) using these new evolutionary markers. Based on the most recent findings by Pardiñas et al. (2018), we hypothesized that the risk alleles of schizophrenia underwent negative selection after modern humans branched away from Neanderthals and Denisovans.

## Materials and Methods

### Data Sources

#### GWAS

Summary statistics of GWAS SNPs were obtained from the PGC schizophrenia study^1^, which consisted of 34,241 cases and 45,604 controls.

#### MAF

Minor allele frequency (MAF) information from the 1000 Genomes Project in European (pop_id = 16652) populations were downloaded from the dbSNP149 database^2^.

#### Human-Specific Sites

General information on MD/AD sites were downloaded from the Max Planck Institute’s Evolutionary Anthropology website^3^. We have extracted information (NCBI identifier, genome coordinates and ancestral allele of the site) for SNPs within modern-human (MD-SNPs), and archaic-human (AD-SNPs) specific sites. Although most of these sites were fixed in modern humans and did not have alternative alleles, 91,752 MD-SNPs (28.5%) and 66,952 AD-SNPs (31.0%) were identified in the PGC schizophrenia GWAS following cross-table querying using NCBI identifiers (rsID) or chromosome coordinates as keys. It was these polymorphic sites that were used in the subsequent analyses (Supplementary Figure S1).

### Analytical Approach

#### Linkage Disequilibrium-Pruning Approach

Prior to statistical analysis, available SNPs were subjected to a linkage disequilibrium (LD)-based SNP pruning process because statistical tests, as described below, assume independence of the studied data. The pruning process was conducted by PLINK software in a 1 Mb window in which any pair of SNPs with *R*^2^ > 0.2 was noted and SNPs were greedily pruned from the window until no such pairs remained. During the pruning process, SNPs were randomly removed with the same priority. The 1000 genome project phase 3 data^4^ were used as a reference in the pruning process.

**Table 1:** Schematic illustration of *F*-score.

	Within all genome regions	Within MD/AD sites	Proportion
All available SNPS	a	b	b/a (expected)
SNPs within the queried *p*-value bin	c	d	d/c (observed)

#### Enrichment Analysis of Schizophrenia SNPs for Human-Specific Sites

To control the potential bias caused by MAF, only SNPs with a MAF < 0.1 were included in the enrichment analysis. The MAF of <0.1 was selected because variants in human-specific sites occur at this frequency or below. Fold change scores (*F*-scores) within each association *p*-value decile bin (p ∼ [1, 0.886], [0.886, 0.781], [0.781, 0.671], [0.671, 0.559], [0.559, 0.443], [0.443, 0.336], [0.336, 0.233], [0.233, 0.140], [0.140, 0.054], and [0.054, 0]) were calculated as the difference between the observed proportion and the expected proportion:

$$\mathrm{F score}=\frac{b\times c}{a\times d}$$

where the observed proportion is the ratio of the distribution of SNPs within the queried *p*-value bin located in MD/AD sites (d in Table 1), to the distribution of these SNPs in all regions of the genome (c). Whereas, the expected proportion is the ratio of the distribution of all available SNPs located in MD/AD sites (b), to the distribution of these SNPs in all regions of the genome (a). The Fisher’s exact test was used to quantify the difference between AD and MD sites within each decile bin.

#### Identification of Derived-Risk or Derived-Protective Alleles

To further investigate changes of risk and protective alleles during the process of human evolution, we identified the derived-risk and derived-protective alleles for schizophrenia. Risk and protective alleles for schizophrenia were determined using summary results from the PGC GWAS (Schizophrenia Working Group of the Psychiatric Genomics Consortium, 2014). Derived/ancestral alleles were identified using the chimpanzee genome as a reference. Those SNPs within MD/AD sites were divided into the derived-risk category, in which the derived allele is the risk allele for schizophrenia (the ancestral allele is the protective allele), and the derived-protective category, in which the derived allele is the protective allele for schizophrenia. We then calculated the ratio of derived-risk and derived-protective schizophrenia SNPs in each of the decile *p*-value bins described above to examine the pattern of risk and protective allelic substitutions during the recent evolution of humans. The Fisher’s exact test was used to identify the statistical significance within each of the decile bins. All statistical tests have been performed in the R program v3.2.3.

#### Cross-Disorder Analyses

To determine if our results observed in schizophrenia could also be observed in other psychiatric disorders, we obtained PGC GWAS summary results^1^ for bipolar, autism and major depressive disorder. The same analytical pipeline used to examine the schizophrenia data (described above) was applied separately to the bipolar, autism and major depressive disorder GWAS data. The chromosome coordinates for genome build 38 (hg38) and build 18 (hg18) were aligned with the coordinates for genome build hg19 by the LiftOver software, along with corresponding conversion references^5^^6^.

## Results

### Enrichment Analysis of Schizophrenia SNPs

**FIGURE 2:**
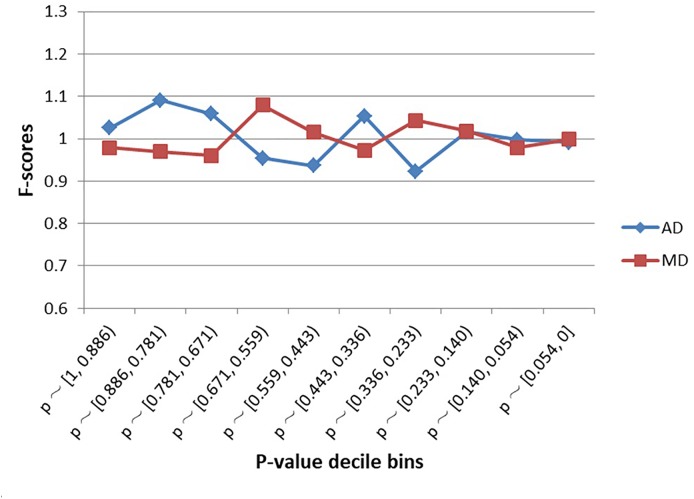
Enrichment (*F*-scores) of schizophrenia SNPs for MD and AD sites by *p*-value. The red square and blue diamond represent the *F*-scores of MD and AD sites among the examined schizophrenia GWAS SNPs. MD = Modern-human-specific sites; AD = Archaic-human-specific sites.

As shown in Figure 2, SNPs examined in the schizophrenia GWAS were not significantly enriched within MD sites or AD sites, regardless of decile bin (Supplementary Table S1). Furthermore, there was no difference in the proportion of MD-SNPs (overall *p*-value across all bins = 0.66) or AD-SNPs (*p*-value = 0.56) among all GWAS SNPs.

### Schizophrenia Risk and Protective Allelic Substitution

**FIGURE 3:**
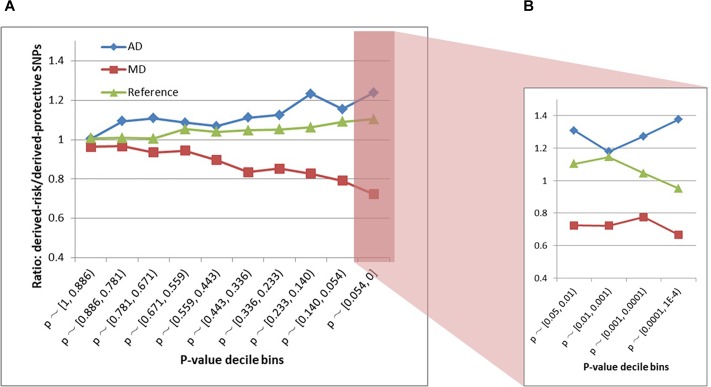
**(A)** Derived-risk/derived-protective allele ratios within AD and MD sites. **(B)** An expanded view of the *p* < 0.054 bin. MD = Modern-human-specific sites; AD = Archaic-human-specific sites.

The schizophrenia SNPs within MD and AD sites had diametrically opposite evolutionary patterns (Figure 3A,B and Supplementary Table S2). The AD sites contained more derived-risk alleles for schizophrenia compared with the MD sites, whereas the MD sites had more derived-protective alleles. The strongest difference (*p*-value = 3.9 × 10^−15^) was found within the decile bin containing SNPs with the smallest *p*-value in the PGC schizophrenia GWAS (Supplementary Table S2).

### Cross-Disorder Analysis

Similar to schizophrenia, SNPs from the bipolar, autism and major depressive disorder GWAS were not significantly enriched within MD sites or AD sites, regardless of the decile bin examined (Supplementary Figure S2). In contrast, we did not detect a similar evolutionary pattern as was observed in schizophrenia (Supplementary Figure S3 and Table S3).

## Discussion

Our findings show that since the modern human lineage split from Neanderthals and Denisovans, risk alleles for schizophrenia but not for other psychiatric disorders, have been progressively eliminated from the modern human genome. Interestingly, the tendency toward eliminating risk and retaining protective alleles has been identified in not only nominally associated SNPs, but also SNPs that currently have not been associated with schizophrenia (i.e., SNPs with *p* values > 0.05). One explanation for this observation is background selection. Background selection is based on the notion that negative selection could decrease the frequency of a deleterious allele, along with the removal of linked variation within the same LD block. Based on background selection, the elimination of schizophrenia risk alleles may not be the result of their intrinsically deleterious effects, but the negative selection of causal alleles.

The enrichment of schizophrenia SNPs in pHAR regions and NSS regions was identified by Xu et al. (2015) and Srinivasan et al. (2016), respectively. Srinivasan attributed their observation to the effect of positive selection after the divergence of humans and Neanderthals. However, the most recent study by Pardiñas et al. (2018) has emphasized the role of background selection in the persistence of risk alleles for schizophrenia. Contrary to the perspective in Srinivasan’s study, Pardiñas et al. (2018) suggested that SNPs under positive selection are less likely to be associated with schizophrenia. Our findings are consistent with those reported by Pardiñas et al. (2018) in that our results support negative selection and corresponding background selection of schizophrenia risk alleles rather than positive selection.

**FIGURE 4:**
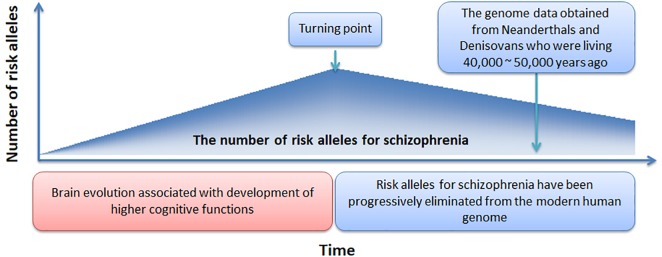
A novel evolutionary framework for the genetic origin of schizophrenia.

In Figure 4, we offer a simple preliminary framework that integrates our results within an evolutionary context. Our framework adopts the by-product hypothesis’ notion that the number of schizophrenia risk alleles increased with the development of the social brain, language, and high-order cognitive functions (Crow, 2000; Burns, 2004). Aligned with this notion, we speculate that around 100,000 – 150,000 years ago (Burns, 2004), before the migration of modern humans out-of-Africa (Stringer and Andrews, 1988), there was a “turning point” at which time the number of schizophrenia risk alleles plateaued. Thereafter, risk alleles for schizophrenia have been progressively but slowly eliminated from the modern human genome while undergoing negative selection pressure.

Support for our proposed framework would ideally involve evidence suggesting progressive reductions in schizophrenia incidence over the past 100,000 – 150,000 years, along with evidence showing greater schizophrenia polygenic burden among our more distant human ancestors. However, currently we are limited to DNA obtained from Neanderthals and Denisovans. In addition, the calculation and comparison of schizophrenia polygenic burden in Neanderthals and Denisovans with that observed in modern humans would be an effective approach to validate the proposed framework. However, the time-frame by which human evolution occurred (e.g., >million years) and the relatively recent operationalization of schizophrenia, pose a significant challenge in evaluating changes in the incidence of schizophrenia from an evolutionary perspective. However, an epidemiological study has suggested the incidence of schizophrenia is declining (McGrath et al., 2008).

Our framework could be strengthened or refined by answers to several outstanding questions. First, when did the “turning point” occur? We have speculated the occurrence of this event to have taken place 100,000 – 150,000 years ago but more precise estimates would allow for more sophisticated evolutionary models to be created. Second, how many schizophrenia common risk alleles were present at the turning point? Our framework assumes the number of schizophrenia risk alleles or polygenic burden was greater among our human ancestors but the extent of this additional burden is unknown. Third, what is the rate at which common risk alleles have been eliminated and to what extent have other evolutionary mechanisms such as balancing selection or sexual selection counteracted the rate of allele elimination? Our proposed framework assumes removal of risk alleles has occurred in a static, linear fashion since the turning point. However, to confirm this assumption, DNA from more distant ancestors will be required. Finally, can a single evolutionary framework explain the genetic origin of schizophrenia? Our analysis and framework assume that schizophrenia is a unitary disorder. However, it is widely accepted that schizophrenia represents a clustering of various symptoms rather than a unitary disorder and any comprehensive framework is likely to require a combination of models. As such, our analyses would have ideally been performed on more homogenous populations that shared similar symptoms. Unfortunately, most public schizophrenia GWAS datasets are limited in the amount of symptom level data available, prohibiting these types of analyses. Nevertheless, our findings suggest that risk alleles for schizophrenia have been progressively eliminated from the modern human genome, regardless of the presumed symptom heterogeneity within our sample. Future investigations of schizophrenia GWAS data with high quality phenotyping is warranted.

Despite the novelty and strength of our study, we acknowledge several limitations. Due to the limited number of associated SNPs, the study did not examine the enrichment and substitution of schizophrenia susceptibility under strict *p*-value thresholds. Novel evolutionary markers encompassing more schizophrenia SNPs are therefore required to further investigate SNPs with genome-wide significance. Second, insertion-deletion (indels) variants were not included in our analysis due to the low number available in our dataset. Indels play regulatory roles in brain functions, thus future studies should explore their contribution to the genetic origins of schizophrenia. Third, our findings rely on genome information of several archaic humans, but the psychiatric status of the Neanderthal or Denisovan individuals remains unknown. If any of them were affected by psychosis, our findings could be biased. Finally, other evolutionary models, such as the sexual selection and balancing selection model (Nettle, 2001; Del Giudice, 2017), have been proposed to reconcile the evolution paradox in schizophrenia. However, the present study did not empirically evaluate these models because evolutionary markers available are not suitable for testing such evolutionary models.

In sum, we have performed a novel evolutionary analysis using schizophrenia and other psychiatric disorder GWAS data and comparative genome results in modern and archaic humans. Our study, for the first time, provides experimental evidence supporting the role of negative selection in eliminating risk alleles for schizophrenia but not other psychiatric disorders from the modern human genome. Based on these theoretical and biological findings, we have proposed a novel evolutionary framework to stimulate further research on the evolutionary paradox and genetic origin of schizophrenia.

## Author Contributions

CL conceived and designed the study, performed bioinformatics and statistical analyses. CB and CL interpreted the main findings. CB, IE, and CP supervised the work.

## Conflict of Interest Statement

The authors declare that the research was conducted in the absence of any commercial or financial relationships that could be construed as a potential conflict of interest.

## Footnotes

1 https://www.med.unc.edu/pgc/results-and-downloads

2 https://ftp.ncbi.nih.gov/snp/

3 http://cdna.eva.mpg.de/neandertal/

4 http://phase3browser.1000genomes.org/index.html

5 https://genome.ucsc.edu/cgi-bin/hgLiftOver

6 http://hgdownload.soe.ucsc.edu/goldenPath/hg38/liftOver/
