# Supplementary material for: Interrogating the Evolutionary Paradox of Schizophrenia: A Novel Framework and Evidence Supporting Recent Negative Selection of Schizophrenia Risk Alleles
Source: Front Genet. 2019 Apr 30;10:389. doi: 10.3389/fgene.2019.00389 (PMC6502987; doi:10.3389/fgene.2019.00389)
Supplement: Supplementary file 1 [file Data_Sheet_1.docx]

**SUPPLEMENTARY MATERIAL**

**Interrogating the evolutionary paradox of schizophrenia: A novel framework and evidence supporting recent negative selection of schizophrenia risk alleles**

**Table of Contents**

**Supplementary Figures**

Figure S1. Workflow of the present study 2

Figure S2. Enrichment (F-scores) of GWAS SNPs for MD and AD sites by p-value 3

Figure S3. Derived-risk/derived-protective allele ratios in psychiatric disorders 4

**Supplementary Tables**

Table S1. Enrichment analysis of schizophrenia SNPs 5

Table S2. Distribution of derived-risk and derived-protective SNPs in decile groups created based on p value 6

Table S3. Distribution of derived-risk and derived-protective SNPs that are nominally associated with different psychiatric disorders 7

**Supplementary Figures**


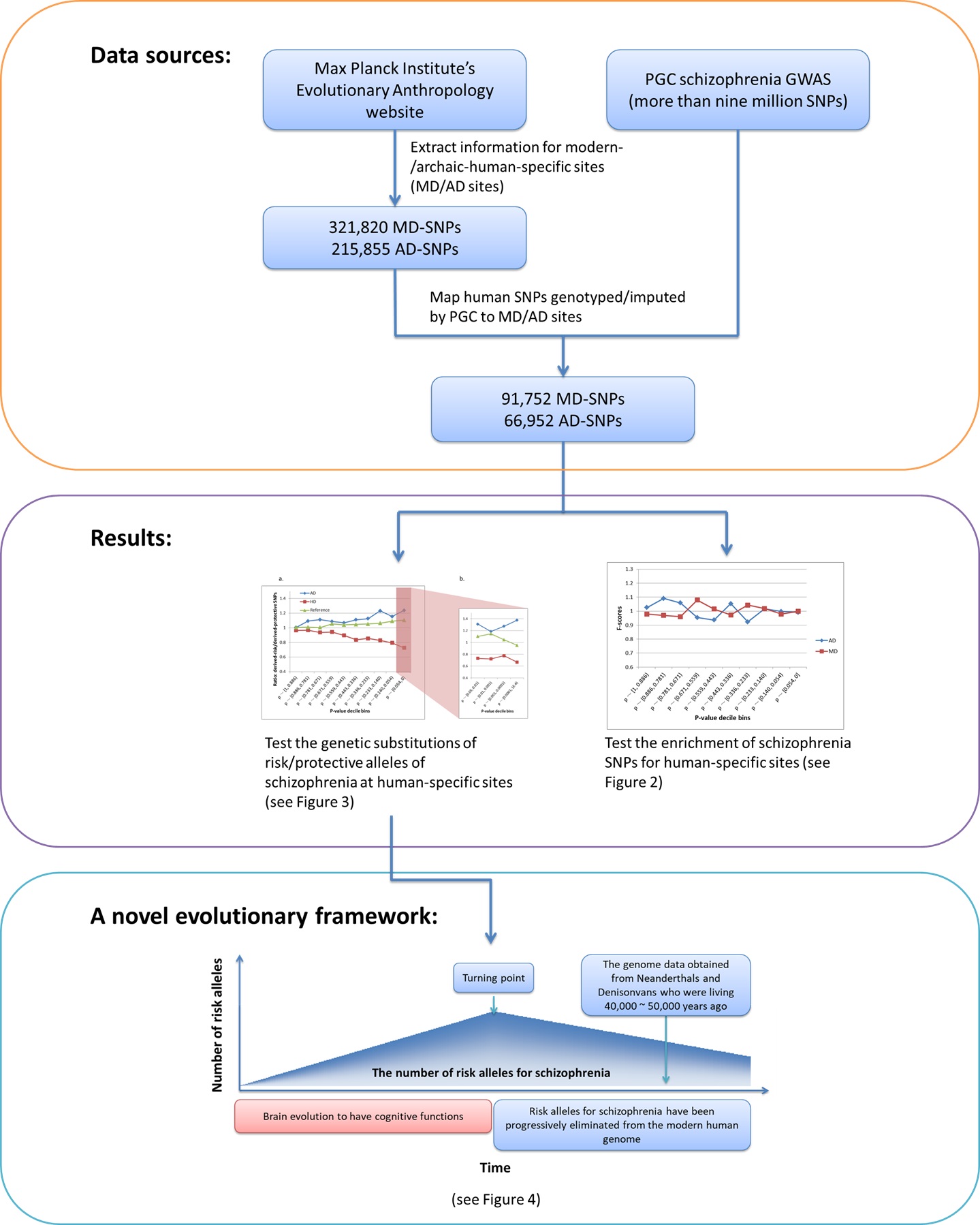


Supplementary Figure 1. Workflow of the present study. PGC = Psychiatric Genomics Consortium; MD = Modern-human-specific sites; AD = Archaic-human-specific sites; SNPs = single nucleotide polymorphisms.


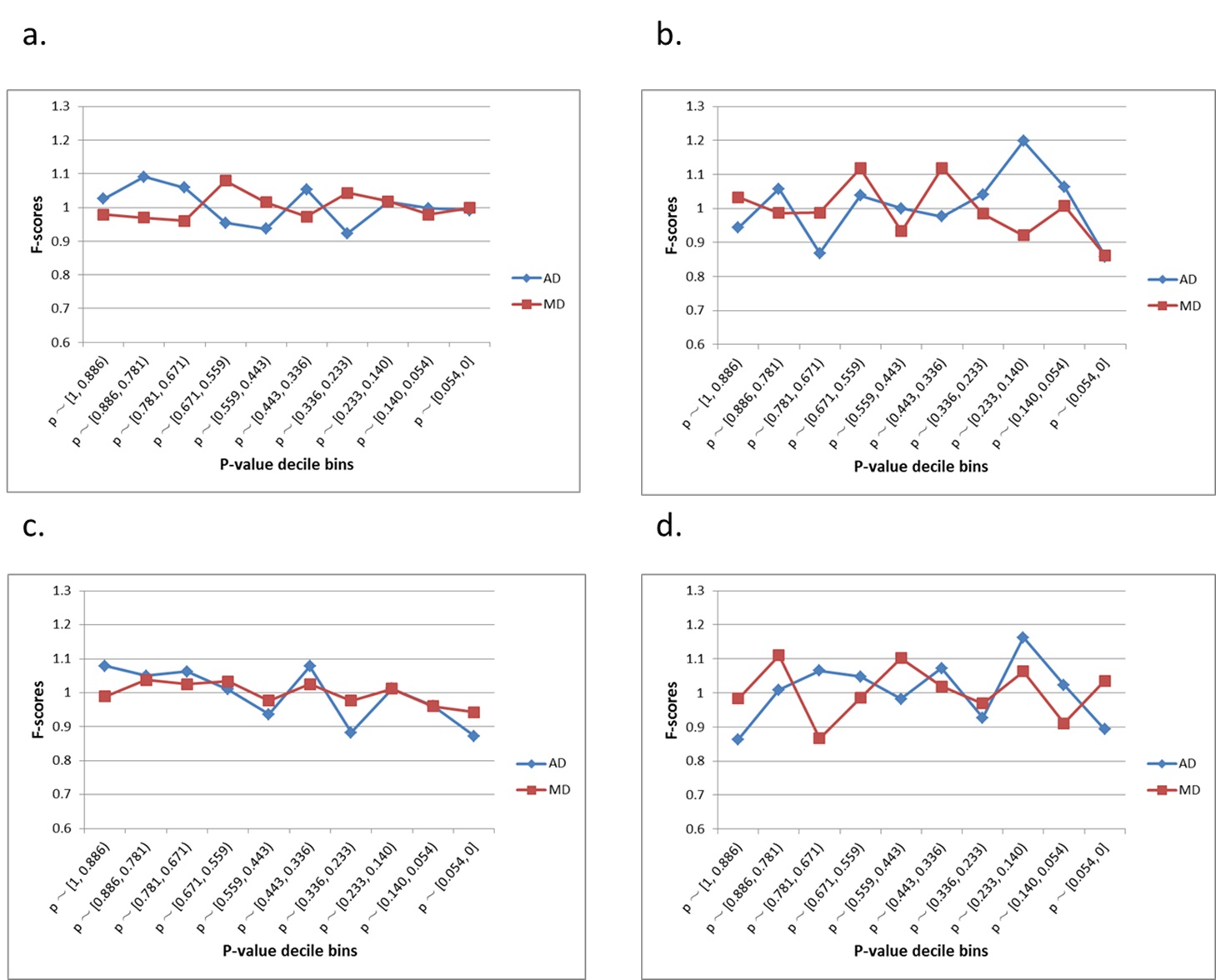


Supplementary Figure 2. Enrichment (F-scores) of GWAS SNPs for MD and AD sites by p-value. (a) schizophrenia, (b) major depressive disorder, (c) autism, (d) bipolar. The red square and blue diamond represent the F-scores of MD and AD sites among the examined GWAS SNPs. MD = Modern-human-specific sites; AD = Archaic-human-specific sites.


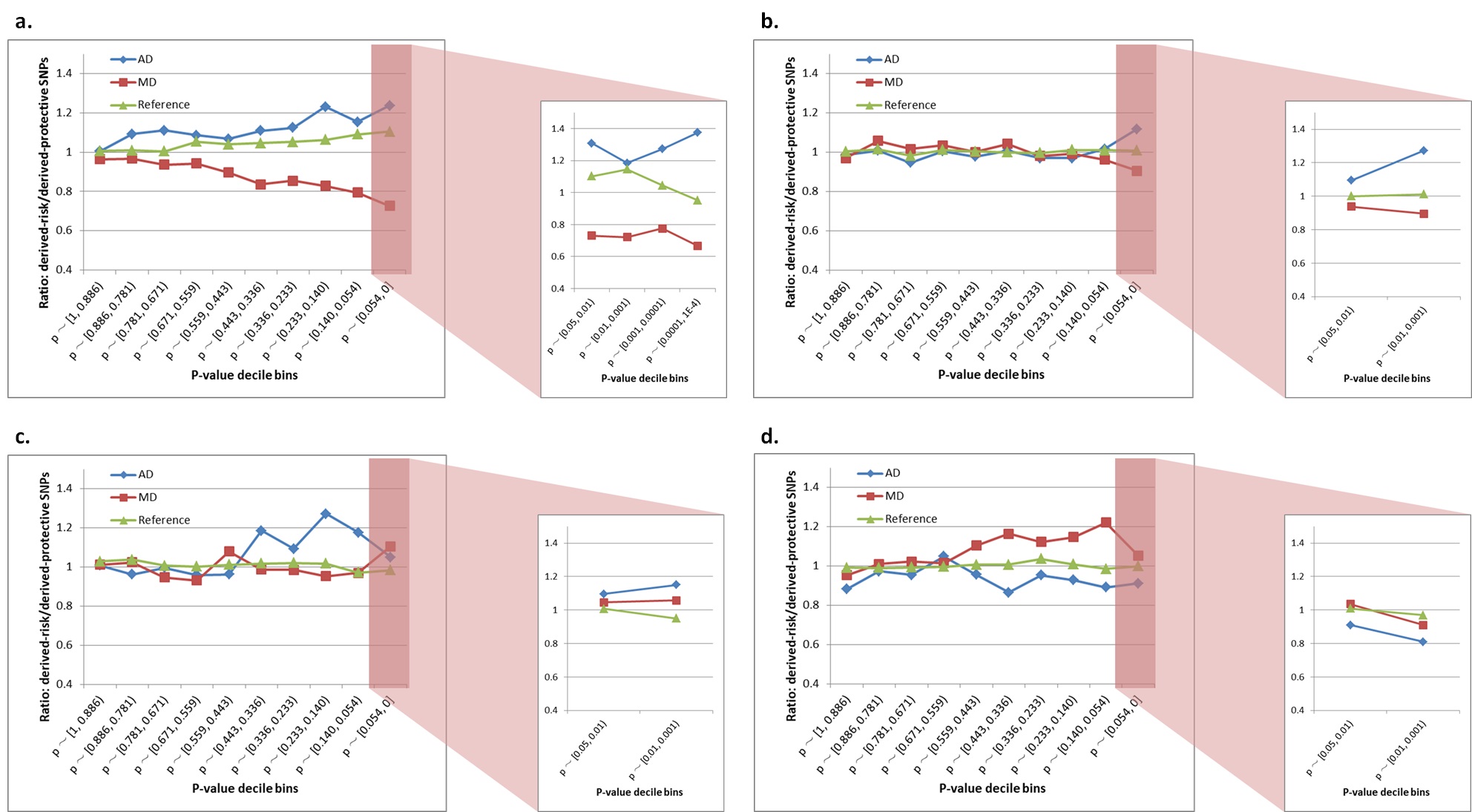


Supplementary Figure 3. Derived-risk/derived-protective allele ratios in **(a)** schizophrenia, **(b)** autism, **(c)** major depressive disorder and **(d)** bipolar disorder. The red square and blue diamond represent the F-scores of MD and AD sites among the examined GWAS SNPs. MD = Modern-human-specific sites; AD = Archaic-human-specific sites.

**Supplementary Tables**

Supplementary Table 1. Enrichment analysis of schizophrenia SNPs

| p-value bins | SNPs | SNPs in AD sites | SNPs in MD sites | Fisher’s p (AD) | Fisher’s p (MD) |
| --- | --- | --- | --- | --- | --- |
| p ～ [1, 0.886) | 29929 | 297 | 517 | 0.68 | 0.67 |
| p ～ [0.886, 0.781) | 27375 | 289 | 468 | 0.16 | 0.53 |
| p ～ [0.781, 0.671) | 28869 | 296 | 489 | 0.34 | 0.41 |
| p ～ [0.671, 0.559) | 29669 | 274 | 565 | 0.47 | 0.08 |
| p ～ [0.559, 0.443) | 31217 | 283 | 559 | 0.31 | 0.73 |
| p ～ [0.443, 0.336) | 29305 | 299 | 503 | 0.38 | 0.57 |
| p ～ [0.336, 0.233) | 29447 | 263 | 542 | 0.22 | 0.35 |
| p ～ [0.233, 0.140) | 27964 | 275 | 502 | 0.79 | 0.70 |
| p ～ [0.140, 0.054) | 27151 | 272 | 486 | 1 | 0.66 |
| p ～ [0.054, 0] | 22831 | 219 | 402 | 0.94 | 1 |

Supplementary Table 2. Distribution of derived-risk and derived-protective SNPs in decile groups created based on p value.

| p-value bins | AD sites | | |  | MD sites | | | p value |
| --- | --- | --- | --- | --- | --- | --- | --- | --- |
|  | Derived-risk | Derived-protect | Ratio |  | Derived- risk | Derived-protect | Ratio |  |
| p ～ [1, 0.886) | 1295 | 1290 | 1.004 |  | 1898 | 1971 | 0.963 | 0.4163 |
| p ～ [0.886, 0.781) | 1278 | 1169 | 1.093 |  | 1700 | 1759 | 0.966 | 0.02011 |
| p ～ [0.781, 0.671) | 1322 | 1192 | 1.109 |  | 1733 | 1852 | 0.936 | 0.001146 |
| p ～ [0.671, 0.559) | 1267 | 1166 | 1.087 |  | 1789 | 1896 | 0.944 | 0.007134 |
| p ～ [0.559, 0.443) | 1289 | 1207 | 1.068 |  | 1759 | 1964 | 0.896 | 0.0006998 |
| p ～ [0.443, 0.336) | 1256 | 1130 | 1.112 |  | 1558 | 1866 | 0.835 | 9.38E-08 |
| p ～ [0.336, 0.233) | 1186 | 1054 | 1.125 |  | 1582 | 1855 | 0.853 | 3.75E-07 |
| p ～ [0.233, 0.140) | 1152 | 934 | 1.233 |  | 1390 | 1681 | 0.827 | 2.31E-12 |
| p ～ [0.140, 0.054) | 1047 | 906 | 1.156 |  | 1267 | 1602 | 0.791 | 1.24E-10 |
| p ～ [0.054, 0] | 806 | 651 | 1.238 |  | 882 | 1221 | 0.722 | 3.85E-15 |
| p ～ [0.05, 0.01) | 608 | 465 | 1.308 |  | 655 | 905 | 0.724 | 1.40E-13 |
| p ～ [0.01, 0.001) | 199 | 169 | 1.178 |  | 217 | 300 | 0.723 | 0.0003864 |
| p ～ [0.001, 1E-4) | 42 | 33 | 1.273 |  | 45 | 58 | 0.776 | 0.1291 |
| p ～ [1E-4, 1E-5) | 11 | 8 | 1.375 |  | 14 | 21 | 0.667 | 0.2593 |

The sum numbers of derived-risk and derived-protective SNPs were not equal among groups because the LD-pruning process removed more linkage SNPs when a stricter p-value threshold was applied.

Supplementary Table 3. Distribution of derived-risk and derived-protective SNPs that are nominally associated with different psychiatric disorders.

|  | MD sites | | |  | AD sites | | | p value |
| --- | --- | --- | --- | --- | --- | --- | --- | --- |
|  | Derived-risk | Derived-protect | Ratio |  | Derived-risk | Derived-protect | Ratio |  |
| Schizophrenia | 882 | 1221 | 0.722 |  | 806 | 651 | 1.238 | 3.85E-15 |
| Bipolar | 343 | 326 | 1.052 |  | 184 | 202 | 0.911 | 0.277 |
| Major depressive disorder | 188 | 170 | 1.106 |  | 106 | 101 | 1.05 | 0.794 |
| Autism | 502 | 535 | 0.905 |  | 360 | 329 | 1.118 | 0.128 |
